# Common Fatal Neurodegenerative Diseases Revisited: Beyond Age, Comorbidities, and Devastating Terminal Neuropathology There Is Hope With Prevention

**DOI:** 10.3389/fneur.2022.901447

**Published:** 2022-05-11

**Authors:** Lilian Calderón-Garcidueñas

**Affiliations:** ^1^The University of Montana, Missoula, MT, United States; ^2^Universidad del Valle de México, Ciudad de México, Mexico

**Keywords:** air pollution, quadruple aberrant pathology, preclinical diagnosis, nanoparticles, Alzheimer's, Parkinson's, TDP-43 pathology, children and young adults

## Introduction

Alzheimer's and Parkinson's diseases (AD, PD), fronto-temporal dementia (FTD), and amyotrophic lateral sclerosis (ALS) have caused deaths of thousands of Americans, and the number of patients will continue to increase in the next few decades. Ethnic variations, country/city residency, and targeted occupational high-risk backgrounds are an indication that complex environmental and genetic risk factors play a significant role. Few people will deny that air pollution causes neuroinflammation and oxidative stress, while fine particulate matter (PM_2.5_), ozone (O_3_), nitrogen dioxide (NO_2_), and other pollutants are associated with several types of dementia and PD (1–5). It is also clear that psychiatric morbidity, including depressive symptoms and suicide, are associated with exposures to NO_2_ and PM_2.5_ (6, 7), and traffic pollution contributes to oxidative stress and inflammation in exposed newborns, negatively impacts executive function, may increase the risk of attention-deficit/hyperactivity and Autistic Spectrum Disorders and reduces cortical thickness primarily in sensorimotor brain regions (8–13).

The public health impact of air pollutants starts *in utero* and it cannot be ignored (9, 14). Maitre et al. (14) have shown that smoking and car traffic exposures during pregnancy had the strongest associations with behavioral scores (e.g., smoking with ADHD index, aMR:1.31 [1.09; 1.59]). Air pollution impacts our health from intrauterine life and thus the finding of highly reactive and toxic solid nanoparticles (NPs) in fetal human brains at postconceptional weeks PCW 8–15. Moreover, NPs in maternal and fetal placental compartments suggests that the placental barrier is not limiting the access of environmental NPs (15). Fetal brain combustion and industrial NPs causing subcellular neural and endothelial changes raise medical concerns, including neurological and neurodegenerative lifelong consequences.

This begs the question: “How do we handle as health providers, the presence of quadruple aberrant proteins- hallmarks of AD, PD, FTD, and ALS- in the brains of children and young adults highly exposed to NPs? What could we tell the parents of young urbanites with behavioral problems? Or teachers asking about poor academic performance across entire classrooms? Or the young woman with rapid eye movement sleep behavior disorder (RBD)? (16–19).

## Quadruple Aberrant Proteins in Highly Exposed Urbanites Children and Young Adults

In neuropathology, the diagnosis of major neurodegenerative diseases is based on the presence of specific abnormal proteins, in vulnerable cells, involving at different paces, multiple neurotransmitter systems as in Parkinson's disease where the earliest lesions develop at non-nigral sites, the olfactory bulb, and the enteric nervous system (ENS) (20). In the sporadic form of Alzheimer's disease (sAD) accounting for 95% of AD cases, neuropathologists have hypothesized tau pathology within select projection neurons with susceptible microenvironments can initiate sAD (21). Transactivation response DNA binding protein 43 kDa (TDP-43)-normally a nuclear protein-, becomes a key pathologic protein in ALS and FTD. Neurons and glial cells in ALS, FTD, and the amyotrophic lateral sclerosis-frontotemporal spectrum disorder (ALS-FTSD) exhibit nuclear TDP-43 mislocalization, and cytoplasmic inclusions (22–25). ALS is associated with a spectrum of clinical phenotypes, with cognitive and/or behavioral symptoms and shows progressive degeneration of upper and/or lower motor neurons (26). Interestingly, PD features have been reported in up to 30% of ALS patients, and Lewy bodies, associated with Lewy body disease (LBD), have been reported in a small number of ALS cases (27). The overlap of misfolded proteins among patients with AD at autopsy - tau and Aβ, α-synuclein, and TDP-43, along with Braak neurofibrillary tangle stages I to VI-, is not unusual. Karanth et al. (28) supported that quadruple misfolded proteins are a common substrate for cognitive impairment and the prevalence of comorbid α-synuclein and TDP-43 with AD pathology (tau and Aβ) complicate efforts to identify therapies to treat and prevent AD.

**Figure 1:**
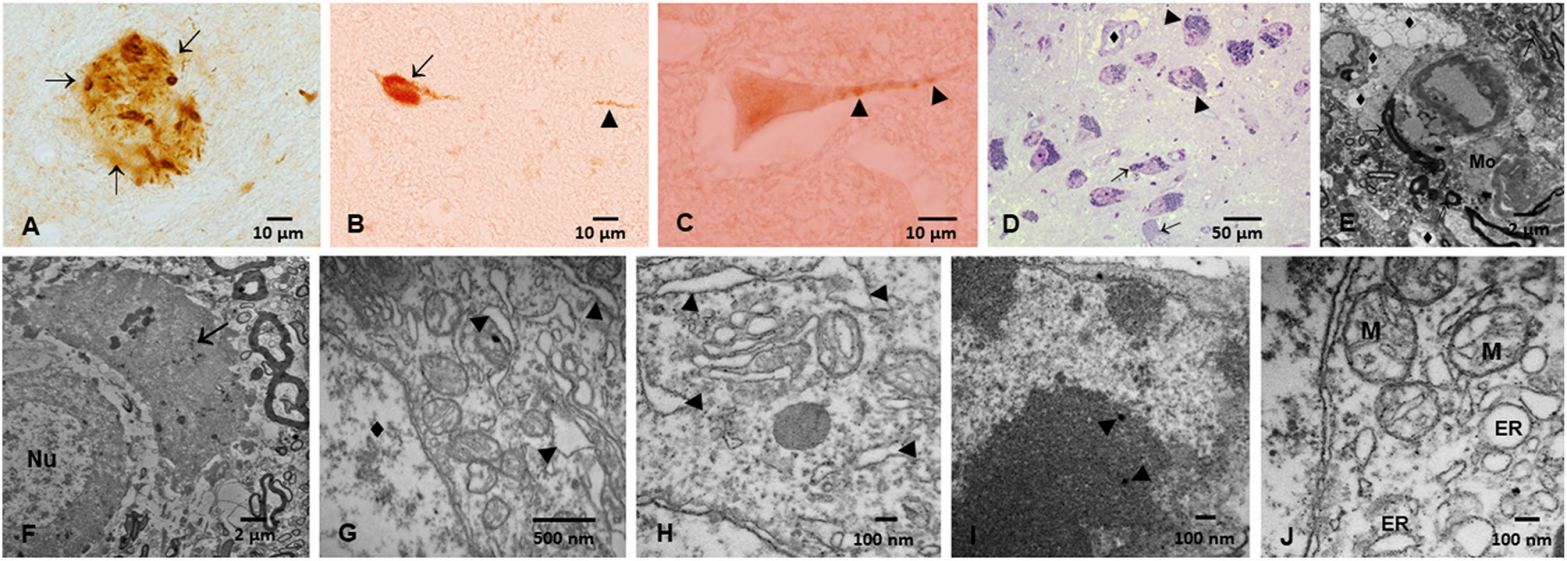
Immunohistochemistry of hyperphosphorylated tau (p-Tau) in substantia nigrae pars compacta (SNpc) and brainstem, and Electron Microscopy in SNpc from Metropolitan Mexico City subjects. **(A)** Hyperphosphorylated tau (p-Tau) mature plaque (arrows) in midbrain, 40y old male. **(B)** Lower medulla p-Tau tangle (short arrow) and a p-Tau positive neurite (arrowhead) in a 13y old girl. **(C)** Raphe neuron with granular positive p-Tau (arrowheads) staining in a 3y old boy. **(D)** SNpc, 1μm toluidine blue section showing neurons with abundant cytoplasmic neuromelanin (NM) (arrowheads) in sharp contrast with neurons with scanty cytoplasm with few NM (short arrows). One small vessel (♦) has a vacuolated perivascular neuropil. **(E)** SNpc neurovascular Unit (NVU). Blood vessels are seen with leaking walls with clusters of lipids in the neuropil. The neuropil is vacuolated (♦). A portion of a macrophage is seen (Mo). **(F)** 11-month old baby. SNpc neuron with a nucleus (Nu) and significant damage to the surrounding neuropil i.e., large vacuolated spaces with debri and macrophage-like cells (short arrow). **(G)** 12-year-old, SNpc neurons with dilated endoplasmic reticulum ER (arrowheads), nucleus marked (♦). **(H)** 26 year old, SNpc dilated ER (arrowheads). **(I)** Nanoparticles inside a SNpc neuronal nucleus (arrowheads) **(J)** SNpc abnormal mitochondria (M) and dilated ER.

We have identified aberrant p-Tau, Aβ, *A* synuclein, and abnormal TDP-43 in Metropolitan Mexico City (MMC) forensic autopsies in residents dying in accidents, homicides, and suicides, age 25.3 ± 9.2 y. These forensic cases had no extra-neural pathology, 99.5% had AD hallmarks, 23% Parkinson's disease (PD) and 18% TDP-43 pathology (Figure 1) (17, 18). Early and progressive neurovascular unit (NVU) damage and extensive organelle abnormalities were associated with metal-rich NPs, making solid nanoparticles a culprit for neural pathology in highly exposed subjects.

## Solid, Highly Reactive Metal and Non-Metal Nanoparticles Reaching Pre and Postnatal Neural and Vascular Cell Targets and Organelles Must Be Included As Potential Effectors in Neurodegeneration and Neuropsychiatric Outcomes, Including Dementia

A common denominator for the significant neuropathology encountered in young urbanites is the solid, highly reactive, combustion, and friction ultrafine particulate matter (UFPM) and industrial nanoparticles. We are vastly exposed to UFPM from fossil and no fossil fuel burning, smog from forest fires, volcanic events, and manufactured NPs from food, construction, electronic, and medical industries. These particles ≤ 100 nm in diameter cross every barrier in the body and reach key brain cell organelles (29).

The key issue: redox-active, strongly magnetic nanoparticles, metal and non-metal, their size and shape, biomolecular corona, surface charge, dynamic magnetic susceptibility, anterograde and retrograde axonal transport capabilities, etc., contribute to ROS generation and extensive NVU, mitochondria, endoplasmic reticulum (ER) and endolysosomal network damage, and are catalysts for protein misfolding, aggregation and fibrillation. NPs neurotoxicity essentially can reproduce the path mechanisms we see in common neurodegenerative diseases, including the aggregation and propagation of neural proteins (30–38).

The problem of NPs crossing the placental barrier at the early stages of fetal development, and the documentation of neurons and primitive glia displaying nuclear, organelle, and cytoplasmic Fe, Ti, and Al alloys, Hg, Cu, Ca, Sn, and Si NPs is a serious medical concern (15). Documentation of NPs in experimental animals shows maternal exposures resulting in severe offspring alterations in neurogenesis and synaptogenesis, perivascular accumulation of β sheet rich proteins, and perivascular ER stress with the accumulation of misfolded proteins in the developing brain (39–41).

## Early Diagnosis of Neurodegenerative Disease Continuum, Rethinking the Definition Of Preclinical Stages, and the Overlapping of Aberrant Neural Proteins

### Preventive Medicine at Work

For the 21.8 million residents in Metropolitan Mexico City (MMC) regularly exposed to fine PM (PM_2.5_) above the US 12 μg/m^3^ annual average standard and to high concentrations of highly toxic NPs (42, 43), the issue is how to protect millions of urbanites from the early development and progression of AD, PD, and TDP43 starting in childhood.

We should start by thinking neurodegenerative processes are a continuum from very young ages (may be even *in utero*), multiple abnormal proteins are at play and there is significant overlapping at neuropathological, clinical, laboratory, and imaging levels. A careful review of the quadruple protein pathology in MMC residents points to the olfactory bulb, the ENS, brainstem, and cortex as regions with significant neuropathology: 202/203 subjects age 25.36 ± 9.23 y., exhibited all AD hallmarks, with tau pre-tangles, neurofibrillary tangles (NFT) Stages I-II, amyloid phases 1–2 by the 2 nd decade. While NFT stages III-V were documented in ~25% of 30–40 y old's. Noradrenergic and dopaminergic nuclei, cochlear, vestibular, hypoglossal, spinal trigeminal, oculomotor, and olfactory nerves exhibit a combination of abnormal aberrant proteins in young urbanites (17, 18, 29).

The concept of AD as defined by the National Institute on Aging and Alzheimer's Association Research Framework (44)—“*Alzheimer's disease is defined by its underlying pathologic processes that can be documented by postmortem examination or in vivo by biomarkers”*—is a welcome biologic construct enabling researchers to add variables to the framework aiming for “*an accurate characterization and understanding of the sequence of events that lead to cognitive impairment.”* However, even with the expansion of the original biomarker matrix toward an ATX(N) system with new candidate biomarkers for additional pathophysiological mechanisms such as neuroimmune dysregulation, synaptic dysfunction, and blood-brain barrier (BBB) alterations (45), it is becoming clear, researchers dealing with severe air pollution exposed populations need to be able to define preclinical and clinical stages in their young populations.

The issue of preclinical stage definition is not easy for youngsters, in fact, is not easy in elderly populations where preclinical stage empirical definition of the Alzheimer's continuum is going on right now as The National Institute on Aging and the Alzheimer's Association published new research criteria defining the Alzheimer's continuum (AC) by the presence of amyloid-β biomarkers (46). Focusing on amyloid-β biomarkers is interesting given that in our studies with subjects ≤ 40 y, pTau is the main and foremost abnormal protein encountered even in toddlers while *A*β phases I and II remain with minimal changes for the first four decades of life (17). So, do we need to change the concept of AD when we talk about air pollution exposed subjects? (44). We also have a significant controversy with the duration of preclinical, prodromal, and dementia stages (47), since we are detecting cognitive deficits in childhood associated with structural brain alterations and progressively worse cognition in young adults with abnormal brain MRIs (48). The estimate of 10 years for the preclinical AD duration, prodromal AD of 4 years, and dementia of 6 years for individuals presenting with preclinical AD at age 70 has nothing to do with our experience.

The need to begin by redefining the concept of preclinical disease is urgent.

Are we expected to use the same biomarkers as in older individuals? We cannot be invasive at all. When do we need to start documentation of cognitive trajectories? What instruments should we use? How could we ID the individuals at preclinical stages and most importantly, how are we going to protect them?

Methodological issues are at hand for the AD and PD clinical trials with elderly populations (49), our situation is much uncertain: we have very young people at high risk because of air pollution.

Are we going to wait for the AD clinical diagnostic criteria? Are we going to witness the inexorable course of these diseases and do nothing? Or are we going to get support to work on early clinical, laboratory non-invasive biomarkers and longitudinal brain MRI studies that will allow us to have early interventions before the neurodegenerative processes are advanced and irreversible?

Is there any interest in doing preventive medicine at all?

As research continues to answer the myriad of questions about NPs impact on neural tissues, the precautionary principle should call to accelerate and expand policy on early diagnosis and interventions to abate or eliminate the developing neurodegenerative diseases associated with pollutants.

There is a critical opportunity for early intervention; we should define the link between the pathological cascade of AD, PD, FTD, and ALS and the emergence of early biomarkers in a population with no associated morbidities.

We definitely have current biomarkers limitations in the diagnosis of AD and other major neurodegenerative diseases (50, 51) and their overlap cannot be ignored because the prevalence of AD with α-synuclein and TDP-43 will most certainly complicate efforts to identify therapies to treat lethal diseases as clearly stated by Karanth et al. (28).

We should work on conceptual frameworks and operational research criteria, based on air pollutant scientific evidence to test the hypothesis with longitudinal clinical research studies. The goal is to advance the study of early neurodegenerative diseases, and ultimately, aid the field in moving toward earlier intervention at stages that may be most effective.

It is absolutely no doubt that we should have a clear understanding of how to protect our populations before disclosure of aberrant protein pathology as discussed by van der Schaar et al. (52).

For a megacity, the size of MMC and 21.8 million exposed subjects, reductions in PM_2.5_ emissions, including poorly regulated heavy-duty diesel vehicles should be implemented. We know human exposure to particulate matter pollution damages the brain and UFPM and NPs have to be part of the research frame. Validation of biomarkers that allow the identification of young urbanites at risk for developing neurodegenerative diseases is a currently unmet need. Neurodegenerative diseases are fatal and we have no disease-modifying therapies.

Prevention should be the goal.

## Author Contributions

The author confirms being the sole contributor of this work and has approved it for publication.

## Conflict of Interest

The author declares that the research was conducted in the absence of any commercial or financial relationships that could be construed as a potential conflict of interest.

## Publisher's Note

All claims expressed in this article are solely those of the authors and do not necessarily represent those of their affiliated organizations, or those of the publisher, the editors and the reviewers. Any product that may be evaluated in this article, or claim that may be made by its manufacturer, is not guaranteed or endorsed by the publisher.
